# Is panic disorder a disorder of physical fitness? A heuristic proposal

**DOI:** 10.12688/f1000research.12788.1

**Published:** 2018-03-08

**Authors:** Giampaolo Perna, Daniela Caldirola

**Affiliations:** 1 Department of Clinical Neurosciences, Hermanas Hospitalarias, Villa San Benedetto Menni Hospital, FoRiPsi, Albese con Cassano, Como, Italy; 2 Department of Psychiatry and Neuropsychology, Faculty of Health, Medicine and Life Sciences, Maastricht University, Maastricht, Netherlands; 3 Department of Psychiatry and Behavioral Sciences, Leonard Miller School of Medicine, Miami University, Miami, USA

**Keywords:** panic disorder, heuristic, panic attack

## Abstract

Currently, panic disorder (PD) is considered a mental disorder based on the assumptions that panic attacks (PAs) are “false alarms” that arise from abnormally sensitive defense systems in the central nervous system and that PD is treated with therapies specifically acting on anxiety or fear mechanisms. This article aims to propose an alternative perspective based on the results of some experimental studies. Our heuristic proposal suggests not only that PD may be a mental disorder but also that patients with PD have real abnormal body functioning, mainly involving cardiorespiratory and balance systems, leading to a decline in global physical fitness. PAs, as well as physical symptoms or discomfort in some environmental situations, may be “real alarms” signaling that the adaptability resources of an organism are insufficient to respond appropriately to some internal or external changes, thus representing the transient conscious awareness of an imbalance in body functioning. The antipanic properties of several modern treatments for PD may include their beneficial effects on body functions. Although anxiety or fear mechanisms are evidently involved in PD, we hypothesize that a reduction of physical fitness is the “primum movens” of PD, while anxiety or fear is induced and sustained by repeated signals of impaired body functioning. We propose considering panic in a broader perspective that offers a central role to the body and to contemplate the possible role of somatic treatments in PD.

## Introduction

Panic disorder (PD) is a chronic and disabling condition causing marked distress and deterioration in quality of life, and it often induces benzodiazepine or alcohol abuse and depression ^1, 2^. PD is a complex syndrome that starts with unpredicted panic attacks (PAs), which on recurrence induce subsequent defensive mechanisms, such as anticipatory anxiety and/or maladaptive changes in behavior. Most patients with PD fear or avoid multiple situations in which PAs can occur (i.e. agoraphobia) ^3^. A PA, the core feature of the disorder, is an abrupt surge of somatic symptoms, such as chest pain, palpitations, sensations of shortness of breath, feelings of choking, and dizziness or unsteadiness, accompanied by intense discomfort and/or fear of dying or of losing control. It reaches a peak within minutes and thereafter spontaneously decreases until it disappears.

Presently, PD is conceptualized as a mental disorder and is included in anxiety disorders in both the DSM-5 and the ICD-10 ^3, 4^, based on the two following widely accepted assumptions:

1. PAs are “false alarms” associated with abnormally sensitive defensive systems in the central nervous system ^5^. Although different opinions exist on the type of false alarm, including suffocation false alarm ^6^, inappropriate fear reactions ^7^, or catastrophic misinterpretation of harmless somatic sensations ^8^, there is a general consensus that these alarms are false because patients with PD are physically healthy.
2. PD is successfully treated with therapies affecting anxiety mechanisms and processes, such as psychotropic drugs and/or psychotherapy ^9^.

These two assumptions may not be entirely true. This narrative review aims to suggest a possible alternative perspective based on the results of many experimental studies and our over 25 years of clinical experience with patients with PD.

In our opinion, many findings highlight not only that PD may be a mental disorder but also that patients with PD may have real abnormal or inefficient body functioning, mainly involving cardiorespiratory and balance systems. Consequently, the patient’s physical fitness ( https://www.hhs.gov/fitness/), which is the state of physical wellbeing that allows optimal performance across multiple routine activities ^10^, may be subtly compromised. Physical symptoms and discomfort experienced during PAs and in some environmental situations may be transient but real manifestations of an underlying decline in physical fitness, which becomes apparent under some circumstances.

In the next sections, we detail the alternative explanations for the two assumptions cited above and our personal view on panic. Our suggestion should be considered neither exhaustive nor conclusive, as it is meant to serve as a heuristic proposal with the intent to foster debate and research on this divisive topic.

## Are panic attacks really the results of false alarms?

PD is unique among anxiety disorders because panic symptoms are mainly physical in nature. From a clinical perspective, patients with PD experience somatic symptoms during PAs and complain of several persistent somatic symptoms between PAs during their usual daily life activities, including respiratory difficulties, abnormalities in their heart rate, dizziness, and photophobia. Owing to somatic symptoms, most patients, especially at the onset of the disorder, believe they suffer from a physical disease. After standard clinical examinations and diagnostic procedures, physicians and psychiatrists usually reassure patients that there is nothing wrong with their body functions and ascribe somatic symptoms entirely to an “anxiety state”. However, some findings suggest that panic symptoms may arise from real, subtle alterations of physical functioning in these patients.

Patients with PD are physiologically different from people without PD in several aspects:

1. They have poor cardiorespiratory fitness, as suggested by studies demonstrating poor performance on cardiopulmonary exercise testing ^11, 12^. The reduced fitness in patients with PD does not seem to be related to anxiety variables, such as state and trait anxiety or fear of physical sensations or autonomic arousal ^11, 12^.
2. Although patients do not report any full-blown respiratory disease, they report a higher-than-expected prevalence of obstructive respiratory diseases in childhood, have an irregular breathing pattern when both awake and asleep, exhibit an increased respiratory variability during mild physical activity, have impaired diaphragmatic breathing with reduced vital capacity, and have a condition of chronic hyperventilation ^13– 19^. Furthermore, smoking exacerbates the irregularity in their breathing pattern, whereas smoking does not seem to affect subjects without the disorder similarly ^20^. Patients with PD have behavioral and respiratory hypersensitivity to hypercapnic challenges, with peculiar respiratory patterns during the challenge and recovery phases ^14– 18^. Similarly, they seemingly have a higher sensitivity to other respiratory stimuli, such as hypoxic challenge ^21, 22^ and hyperventilation ^23^. Finally, connections between sleep apnea and PD have been reported ^24^. Overall, although the respiratory system of patients with PD is not so dysfunctional to induce a full-blown respiratory disease, it might be more unstable and sensitive than that of subjects without PD.
3. Although patients do not have any full-blown cardiac disease, they have imbalanced autonomic regulation, reduced heart rate variability, increased time variability of ventricular repolarization, higher regional heterogeneity of ventricular repolarization and atrial depolarization ^25– 28^, and higher variability of ECG-R wave amplitude after beta-adrenergic stimulation with isoproterenol ^29^. PD patients who are normotensive exhibit an impaired circadian blood pressure pattern with an inadequate reduction in nighttime blood pressure (non-dipper pattern) ^30^ as well as an unstable heart rate while asleep ^31^. At least in a certain proportion of patients with PD, PAs may be caused by paroxysmal supraventricular tachycardia ^32^. In addition, these patients exhibit increased arterial stiffness, and several factors negatively affect endothelial function, such as increased homocysteine levels and platelet aggregation or volume and lower levels of nitric oxide ^25– 28^. Once more, these subtle abnormalities can render the cardiovascular system less efficient, and they are considered cardiovascular risk factors. In line with these findings, cardiovascular morbidity and mortality are higher in patients with PD than in the general population, and the association of PD with cardiac disorders has been well documented ^33– 36^.
4. Patients with PD seem to have subtle metabolic disturbances involving acid–base imbalance ^37– 39^.
5. These patients exhibit subclinical abnormalities in their balance system, with postural instability, especially related to impaired visual–vestibular interactions ^40, 41^, as well as consistent rates of abnormal measures under neurotological examination ^42, 43^. In addition, the number of abnormal posturographic scores is correlated with the severity of agoraphobia ^44^. Notably, this link is so close that otoneurologists consider “phobic postural vertigo syndrome”, a clinical condition very similar to panic syndrome. Patients with PD are also photophobic, with a subtle variation of retinal photosensitivity ^45^.
6. Finally, preliminary reports suggest that children at risk of PD have a relative decrease in cardiac vagal function, an irregular breathing pattern, and abnormalities in their saccadic eye movements, which are involved in balance function and responses ^46– 48^.

Overall, these findings suggest that the body physiology in patients with PD is far from being perfect and stable. If patients with PD have subtle abnormalities in their body functioning, it may result in a reduction of global physical flexibility and adaptability to changes. Consequently, PAs may be “real alarms” signaling that something is going wrong in the body and its physiological systems when the adaptability resources of an organism are insufficient to respond appropriately to ongoing internal or external changes. Usually, the physiological processes act outside awareness to maintain homeostasis. We hypothesize that when a “critical threshold” of physical adaptability is exceeded, the dynamic balance breaks down and a transient physical instability may be perceived as an acute conscious experience of physical symptoms and emotional discomfort (i.e. a PA) until the homeostatic balance is restored. Following this concept, as early as 47 minutes before the occurrence of naturally occurring, unexpected PAs, patients with PD display significant patterns of physical instability across many autonomic and respiratory variables, which occur outside of the patient’s awareness ^49^. Thus, a PA may be conceptualized as a “primordial emotion”, i.e. an acute uncomfortable state in which an imbalance in body functions and homeostasis pervades the conscious awareness ^50– 54^. This assertion is in line with the dynamic complex system theory that postulates the existence, in the biological systems, of non-stationary and dynamic balance states, such as homeostatic balance, which may display sudden and radical changes produced by even slight modifications of the biological parameters beyond a certain threshold ^55^. This view may explain (1) the higher sensitivity of patients with PD to multiple physical stimuli, including their hyperreactivity to respiratory stimulation (hypercapnia, hypoxia, or hyperventilation) and peripheral beta-adrenergic stimulation with isoproterenol ^56^, (2) subclinical autonomic hyperreactivity, (3) space and motion discomfort in complex sensorial environments, and (4) nocturnal PAs, which appear as a sudden awakening in a state of panic during non-REM sleep ^57, 58^, a phase in which significant changes in autonomic and respiratory variables occur (e.g. reduction in heart rate and ventilation and increase in carbon dioxide partial pressure [PaCO _2_]) and subcortical homeostatic control of body functions, centered on brainstem and reflex loops, plays a crucial role ^59, 60^.

Finally, we hypothesize that imbalanced body functioning may also underlie some aspects of phobic avoidance. In several daily life situations that patients with PD often fear and/or avoid, such as public transport, open spaces (e.g. marketplaces, bridges, and large roads), enclosed spaces (e.g. shops, theaters, and cinemas), or driving, efficient physical functioning is needed for optimal performance because all of these conditions require multiple and integrated bodily adjustments to changes, involving cardiorespiratory, autonomic, and balance functions. Again, an underlying reduction of physical fitness may induce physically uncomfortable sensations in several environmental situations, which signal a physical inadequacy to bodily changes required by situations. This mechanism can contribute to avoidance behaviors and the occurrence of “expected” PAs in feared situations.

**Figure 1.**
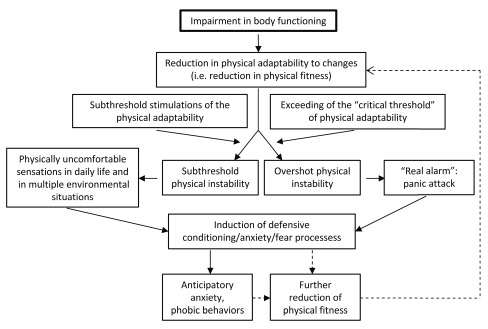
A heuristic model describing hypothesized relationships between the impairment in body functioning and panic disorder.

We underscore that our perspective does not exclude the concept that other mechanisms, more strictly linked to anxiety and fear, are involved in PD. Alarm systems can become hypersensitive over time, and interoceptive or exteroceptive conditioning processes, anticipatory anxiety, and phobic behaviors do exist in patients with PD ^61– 63^; however, we hypothesize that the reduction of physical fitness is the “primum movens” of PD, while anxiety or fear are defensive mechanisms induced and sustained by repeated signals of impaired body functioning ( Figure 1). However, we are aware that patients who experience PAs usually develop attentional bias toward somatic sensations, and, thus, one of the most challenging aspects of our idea is to define the boundaries between “real” somatic signals of body malfunction and heightened awareness of or reactivity to normal somatic sensations due to interoceptive conditioning processes, a key feature of PD ^62– 64^.

Another problematic aspect of our proposal, which deserves consideration, is the possibility that the reduction of physical fitness results from concomitant factors rather than being a specific pathophysiologic feature of PD, including concurrent depression, whose association with autonomic imbalance is well documented ^65^, or unhealthy behaviors of patients with PD, such as avoidance of physical activities ^12^, excessive smoking ^66– 68^, or inappropriate use of alcohol or benzodiazepines ^1, 2^. However, although these factors certainly contribute, it seems unlikely that they can entirely explain the reduced physical fitness of patients with PD. Most of the studies that found abnormalities in body functioning of these patients excluded participants with concomitant depression or alcohol or benzodiazepine misuse and took into account several individual confounding variables. As opposed to patients with PD, patients with major depression are not vulnerable to panic provocation by hypercapnia ^69^, and PD seems to be a risk factor for cardiac diseases independently of depression ^35, 70^. Cigarette smoking generally precedes the onset of panic ^66– 68^ and has a peculiar effect on the respiratory patterns of patients of PD ^20^, suggesting that smoking may contribute to the occurrence of PAs by affecting the vulnerable respiratory function of individuals predisposed to panic. Some studies in healthy first-degree relatives of patients with PD found abnormalities in their body functioning compared to control subjects ^46– 48^, greater likelihood of panic symptoms and respiratory-response abnormalities during hypercapnic challenges ^71– 74^, and a significant relationship between abnormal respiratory response to hypercapnia and later onset of PAs ^75^. Overall, these findings suggest that the reduction of physical fitness may be a specific feature related to panic vulnerability. Finally, on one hand, the physical inactivity surely contributes to the cross-sectional findings of reduced physical fitness in patients with PD, but, on the other hand, these patients might avoid physical exercise precisely because they have an intrinsic impairment in body functioning, which makes physical exercise unpleasant.

The source of impairment in body functioning warrants clarification. We speculate that those circuits that continuously map and modulate the state of the physical configuration of an organism in its multiple, preconscious, biological dimensions to assure optimal body functioning and homeostasis may play a significant role. These circuits include humoral and neural afferent signals from the internal milieu, organs, or peripheral body sites and a set of subcortical and cortical areas, such as the brainstem, hypothalamus, basal forebrain, posterior insula, and medial parietal cortex. These areas dynamically integrate the afferent signals into body maps (conceptualized as “proto-self” by Antonio Damasio) and provide efferent signals to produce body modifications in response to internal or external changes (please refer to 53, 76, 77 for a complete description). Among these areas, the brainstem plays a crucial role. Basic homeostasis, including cardiovascular, respiratory, autonomic, and balance functions, is significantly controlled from the nuclei of the brainstem. In addition, the brainstem is supposed to be the necessary and sufficient platform for the generation of “primordial feelings of the living body” and the basic form of self-consciousness, related to body activities, on which the higher-order of self-awareness and cognitive processes are grounded ^56, 78^. In the brainstem is also where the dorsal periaqueductal gray matter (dPAG) is located, a midbrain area that is thought to play a role in human PAs, based on animal studies demonstrating that it is involved in defensive behaviors triggered by respiratory stimuli and regulated by neurotransmitters implicated in panic pathophysiology ^79– 82^. In patients with PD, recent brain imaging studies have reported structural modifications and peculiar functional responses in the brainstem ^83– 85^, whereas the role of the “fear network” centered on the amygdala ^7^ does not seem consistent ^86^. In addition, the amygdala does not appear to be required in the occurrence of PAs ^87– 89^. Thus, the brainstem may be a suitable neuroanatomical key structure involved in body functioning impairment in PD, possibly through a malfunction of the central control mechanisms of the body’s functions, which becomes evident peripherally with the cardiorespiratory, autonomic, and balance abnormalities in patients with PD, as described earlier. Evidently, the involvement of other different central areas implicated in body regulation, such as the insula, cannot be excluded ^84, 86^. Conversely, the impairment of body functioning may also arise from a primary malfunction in the peripheral physiology of the body, or in peripheral sensors, such as neuroepithelial bodies in the lung ^90^. In turn, peripheral abnormalities may induce adaptive modifications in the central control of body functions, eventually impairing global body functioning. To date, sufficient data to support this possibility do not exist; however, it cannot be excluded.

## Are successful treatments of panic disorder specifically acting on anxiety or fear mechanisms?

Antipanic medications are not simply “psychotropic” medications. The serotonergic system, the main target of the first-line drug treatment of PD (i.e. selective serotonin reuptake inhibitors [SSRIs]), is involved in anxiety or fear mechanisms ^7^. However, it also modulates different brain areas, neural pathways, and peripheral sites directly involved in body functioning ^91– 94^. Hence, SSRIs can act on cardiorespiratory, autonomic, and balance system functions, as described in the following examples:

1. Paroxetine decreases respiratory irregularity in patients with PD ^95^. Respiratory function (forced expiratory volume in 1 second [FEV1], oxygen partial pressure [PaO _2_], and PaCO _2_) and dyspnea improve in patients with chronic obstructive pulmonary disease treated with citalopram ^96^ or sertraline ^97^ as well as in patients with PD treated with antipanic medications ^98^. Paroxetine may be useful in the treatment of obstructive sleep apnea ^99^. The lungs are an essential reservoir of the serotonin transporter ^100^, the target of SSRIs, and this might explain the effect of SSRIs on pulmonary vascular function ^101^.
2. Paroxetine can improve heart rate variability and induce a decrease in the relative cardiac sympathetic activity occurring in patients with PD ^102– 105^. In addition, SSRIs have some beneficial effects on the cardiovascular system, such as inhibiting platelet aggregation, collagen, and thrombin, exerting anti-inflammatory effects, and improving endothelial function ^106^, which may be protective against cardiovascular morbidity in patients with PD.
3. Sertraline reduces the symptoms of paroxysmal hypertension ^107^, a medical condition that can be associated with PD.
4. Seemingly, sertraline and citalopram directly affect cardiac rhythm, at least in animal models, reducing membrane conduction through the inhibition of cardiac Na ^+^ and Ca ^2+^ channels ^108, 109^.
5. Citalopram improves balance system function in patients with PD ^44^, as paroxetine and fluoxetine do in mice ^110^.

Thus, we speculate that antipanic medications might exert their effect by correcting abnormalities of the body’s functions in patients with PD.

Considering non-pharmacological treatments, besides cognitive behavioral therapy, evidence for the therapeutic effects of somatic treatments in PD is growing:

1. In patients with PD, breathing therapies improve panic symptoms ^111, 112^ as well as restore diaphragmatic breathing with regain of vital capacity ^14– 18^; although results are inconclusive, voluntary aerobic physical exercise may provide benefit as an adjunctive strategy for the treatment of PD ^113– 115^.
2. Preliminary unpublished data from our group suggest that vestibular rehabilitation might positively affect panic-phobic symptoms and balance system function in patients with PD and agoraphobia. Likewise, a pilot study reported that subsequent vestibular rehabilitation after cognitive behavioral therapy may significantly improve panic-phobic symptoms in these patients ^116^.

Although we cannot eliminate the notion that these somatic treatments increase PD patients’ perceived sense of control, they could have a beneficial effect on panic symptoms via their influence on body functions. Indeed, a meta-analysis indicated that the combination of exposure, relaxation training, and breathing retraining offers the most consistent evidence for the treatment of PD and is more efficacious than cognitive therapy alone, suggesting that somatic interventions are more effective than the psychological ones, even in psychological treatment packages ^117^.

## Clinical consequences

If PAs result from the activation of “real alarms” related to subtle abnormalities in body functioning, leading to a decrease in physical fitness, we can hypothesize that the latter might be the expression of panic vulnerability. Thus, beyond the full-blown or limited-symptoms PAs, the persistent sensations of physical discomfort in daily life, mainly involving cardiorespiratory and balance systems, might be the active expression of instability or inefficiency of body functioning in these patients. Even when this impairment in body functioning is not adequately intense to trigger a PA, it can signal this risk by inducing physically uncomfortable sensations, thus maintaining anticipatory anxiety and phobic avoidance as active defense mechanisms. Indeed, many patients complain that even when PAs are not present, they feel unfit, with frequent sensations of dyspnea, tachycardia, or dizziness and other unpleasant somatic sensations in their daily life, thereby causing them to feel vulnerable to PAs.

If our hypothesis is correct, the aim of antipanic treatments should be not only the disappearance of PAs but also, most importantly, the achievement of a full sense of physical wellbeing ^118^. Hence, the doses of drug treatments with antipanic agents should be titrated until these subtle physical abnormalities disappear. The future of panic treatment might include the development of somatic treatments that counteract the impairment of body functioning and increase physical fitness, such as breathing training for more harmonic respiration, physical exercise to increase heart rate variability and cardiorespiratory fitness, and vestibular rehabilitation to attain more stable balance system functioning. Indeed, our perspective suggests that the improvement of body functioning might decrease one’s vulnerability to PAs. Following this concept, the improvement of physical fitness might also contribute to decreasing the likelihood of developing PAs in individuals at risk of the disorder, such as the offspring of patients with PD ^46– 48^. Finally, regaining a full sense of physical wellbeing, without any physical discomfort, will also help patients with PD to overcome anticipatory anxiety and agoraphobia during cognitive behavior therapy and to restore full behavioral freedom.

## Conclusion

Our heuristic proposal suggests that the impairment of body functioning, resulting in decreased physical fitness, may play a crucial role in the pathophysiology of PD, being the “primum movens” of the disorder. We do not state that PD is a life-threatening medical disease, or that these patients have unrecognized medical diseases, or exclude the idea that anxiety or fear mechanisms contribute to the development of PD. We propose considering panic in a broader perspective that provides a central role for the body, as clearly patients suggest. Our view may partly explain why many patients with PD do not regain full wellbeing or relapse after standard treatments. Furthermore, it suggests the usefulness of including, in the clinical examination of patients, evaluations of cardiorespiratory patterns and/or balance system functioning to detect possible subtle or subclinical abnormalities. Finally, this perspective may stimulate re-thinking about antipanic treatments and lead to the development of new therapies that consider body functions. We are aware that this view requires confirmation and that more direct experimental evidence is needed. We hope that our considerations help foster debate and research on this controversial topic. As an example, some lines of research may be followed: (1) in patients with PD, testing whether the improvement of cardiorespiratory function by somatic treatments (i.e. physical exercise on a regular basis and breathing training) actually reduces the naturally occurring and hypercapnia-induced PAs; (2) in patients with PD and in subjects at risk of PD, testing, by means of longitudinal studies over the lifespan, (a) the temporal relationships between abnormalities in body functioning and occurrence of PAs and (b) the possible contribution of additional factors, such as unhealthy behaviors and/or depression, to the reduction of physical fitness in patients with PD; (3) in subjects without PD from the general population, testing (a) whether an association exists between decreased levels of cardiorespiratory or balance fitness and subthreshold panic symptoms or vulnerability to hypercapnia-induced PAs and (b) whether pharmacological manipulations aimed at decreasing cardiorespiratory fitness lead to increased vulnerability to hypercapnia-induced PAs.

In conclusion, more attention should be paid to the somatic complaints of patients with PD because these might reflect real subtle abnormalities in their physical fitness, and a more significant role should be given to somatic therapies in the treatment of PD.

More than a century ago, William James stated that bodily sensations “come first” and elicit subsequent feelings and emotions—maybe this is not wrong, at least for patients with PD.

## Abbreviations

PA, panic attack; PaCO _2_, carbon dioxide partial pressure; PD, panic disorder; SSRI, selective serotonin reuptake inhibitor.

## References

[1] Kessler RC Petukhova M Sampson NA: Twelve-month and lifetime prevalence and lifetime morbid risk of anxiety and mood disorders in the United States. *Int J Methods Psychiatr Res.* 2012;21(3):169–84. 10.1002/mpr.1359 22865617 PMC4005415

[2] Batelaan NM Van Balkom AJ Stein DJ: Evidence-based pharmacotherapy of panic disorder: an update. *Int J Neuropsychopharmacol.* 2012;15(3):403–15. 10.1017/S1461145711000800 21733234

[3] American Psychiatric Association: Diagnostic and Statistical Manual of Mental Disorders (Fifth ed.). Arlington, VA: American Psychiatric Publishing;2013 Reference Source

[4] International Classification of Diseases, Tenth Revision (ICD-10). Reference Source

[5] Fava L Morton J: Causal modeling of panic disorder theories. *Clin Psychol Rev.* 2009;29(7):623–37. 10.1016/j.cpr.2009.08.002 19726119

[6] Klein DF: False suffocation alarms, spontaneous panics, and related conditions. An integrative hypothesis. *Arch Gen Psychiatry.* 1993;50(4):306–17. 10.1001/archpsyc.1993.01820160076009 8466392

[7] Gorman JM Kent JM Sullivan GM: Neuroanatomical hypothesis of panic disorder, revised. *Am J Psychiatry.* 2000;157(4):493–505. 10739407 10.1176/appi.ajp.157.4.493

[8] Clark DM Salkovskis PM Ost LG: Misinterpretation of body sensations in panic disorder. *J Consult Clin Psychol.* 1997;65(2):203–13. 10.1037/0022-006X.65.2.203 9086683

[9] Starcevic V: Treatment of panic disorder: recent developments and current status. *Expert Rev Neurother.* 2008;8(8):1219–32. 10.1586/14737175.8.8.1219 18671666

[10] Caspersen CJ Powell KE Christenson GM: Physical activity, exercise, and physical fitness: definitions and distinctions for health-related research. *Public Health Rep.* 1985;100(2):126–31. 3920711 PMC1424733

[11] Caldirola D Namia C Micieli W: Cardiorespiratory response to physical exercise and psychological variables in panic disorder. *Rev Bras Psiquiatr.* 2011;33(4):385–9. 10.1590/S1516-44462011000400013 22189929

[12] Muotri RW Bernik MA: Panic disorder and exercise avoidance. *Rev Bras Psiquiatr.* 2014;36(1):68–75. 10.1590/1516-4446-2012-1012 24604463

[13] Hasler G Gergen PJ Kleinbaum DG: Asthma and panic in young adults: a 20-year prospective community study. *Am J Respir Crit Care Med.* 2005;171(11):1224–30. 10.1164/rccm.200412-1669OC 15764721 PMC2718460

[14] Nardi AE: Panic disorder is closely associated with respiratory obstructive illnesses. *Am J Respir Crit Care Med.* 2009;179(3):256–7. 10.1164/ajrccm.179.3.256a 19158330

[15] Caldirola D Bellodi L Caumo A: Approximate entropy of respiratory patterns in panic disorder. *Am J Psychiatry.* 2004;161(1):79–87. 10.1176/appi.ajp.161.1.79 14702254

[16] Niccolai V van Duinen MA Griez EJ: Respiratory patterns in panic disorder reviewed: a focus on biological challenge tests. *Acta Psychiatr Scand.* 2009;120(3):167–77. 10.1111/j.1600-0447.2009.01408.x 19548964

[17] Grassi M Caldirola D Vanni G: Baseline respiratory parameters in panic disorder: a meta-analysis. *J Affect Disord.* 2013;146(2):158–73. 10.1016/j.jad.2012.08.034 23107756

[18] Grassi M Caldirola D Di Chiaro NV: Are respiratory abnormalities specific for panic disorder? A meta-analysis. *Neuropsychobiology.* 2014;70(1):52–60. 10.1159/000364830 25247676

[19] Stein MB Millar TW Larsen DK: Irregular breathing during sleep in patients with panic disorder. *Am J Psychiatry.* 1995;152(8):1168–73. 10.1176/ajp.152.8.1168 7625465

[20] Caldirola D Bellodi L Cammino S: Smoking and respiratory irregularity in panic disorder. *Biol Psychiatry.* 2004;56(6):393–8. 10.1016/j.biopsych.2004.06.013 15364036

[21] Beck JG Ohtake PJ Shipherd JC: Exaggerated anxiety is not unique to CO _2_ in panic disorder: a comparison of hypercapnic and hypoxic challenges. *J Abnorm Psychol.* 1999;108(3):473–82. 10.1037/0021-843X.108.3.473 10466271

[22] Beck JG Shipherd JC Ohtake P: Do panic symptom profiles influence response to a hypoxic challenge in patients with panic disorder? A preliminary report. *Psychosom Med.* 2000;62(5):678–83. 10.1097/00006842-200009000-00012 11020098

[23] Zugliani MM Freire RC Perna G: Laboratory, clinical and therapeutic features of respiratory panic disorder subtype. *CNS Neurol Disord Drug Targets.* 2015;14(5):627–35. 10.2174/1871527314666150430163142 25924997

[24] Su VY Chen YT Lin WC: Sleep Apnea and Risk of Panic Disorder. *Ann Fam Med.* 2015;13(4):325–30. 10.1370/afm.1815 26195676 PMC4508172

[25] Diveky T Prasko J Latalova K: Heart rate variability spectral analysis in patients with panic disorder compared with healthy controls. *Neuro Endocrinol Lett.* 2012;33(2):156–66. 22592196

[26] Yeragani VK Pohl R Balon R: Twenty-four-hour QT interval variability: increased QT variability during sleep in patients with panic disorder. *Neuropsychobiology.* 2002;46(1):1–6. 10.1159/000063568 12207139

[27] Atmaca M Yavuzkir M İzci F: QT wave dispersion in patients with panic disorder. *Neurosci Bull.* 2012;28(3):247–52. 10.1007/s12264-012-1237-3 22622824 PMC5560325

[28] Yavuzkir M Atmaca M Dagli N: P-wave dispersion in panic disorder. *Psychosom Med.* 2007;69(4):344–7. 10.1097/PSY.0b013e3180616900 17510287

[29] Yeragani VK Pohl R Bär KJ: Exaggerated beat-to-beat R amplitude variability in patients with panic disorder after intravenous isoproterenol. *Neuropsychobiology.* 2007;55(3–4):213–8. 10.1159/000108380 17873495

[30] Alici H Ercan S Bulbul F: Circadian blood pressure variation in normotensive patients with panic disorder. *Angiology.* 2014;65(8):747–9. 10.1177/0003319713512172 24280264

[31] Fisher AJ Woodward SH: Cardiac stability at differing levels of temporal analysis in panic disorder, post-traumatic stress disorder, and healthy controls. *Psychophysiology.* 2014;51(1):80–7. 10.1111/psyp.12148 24102634 PMC3864565

[32] Frommeyer G Eckardt L Breithardt G: Panic attacks and supraventricular tachycardias: the chicken or the egg? *Neth Heart J.* 2013;21(2):74–7. 10.1007/s12471-012-0350-2 23179613 PMC3547429

[33] Walters K Rait G Petersen I: Panic disorder and risk of new onset coronary heart disease, acute myocardial infarction, and cardiac mortality: cohort study using the general practice research database. *Eur Heart J.* 2008;29(4):2981–8. 10.1093/eurheartj/ehn477 18948354

[34] Chen YH Tsai SY Lee HC: Increased risk of acute myocardial infarction for patients with panic disorder: a nationwide population-based study. *Psychosom Med.* 2009;71(7):798–804. 10.1097/PSY.0b013e3181ad55e3 19592516

[35] Gomez-Caminero A Blumentals WA Russo LJ: Does panic disorder increase the risk of coronary heart disease? A cohort study of a national managed care database. *Psychosom Med.* 2005;67(5):688–91. 10.1097/01.psy.0000174169.14227.1f 16204424

[36] Caldirola D Schruers KR Nardi AE: Is there cardiac risk in panic disorder? An updated systematic review. *J Affect Disord.* 2016;194:38–49. 10.1016/j.jad.2016.01.003 26802506

[37] Maddock RJ Buonocore MH Miller AR: Abnormal activity-dependent brain lactate and glutamate+glutamine responses in panic disorder. *Biol Psychiatry.* 2013;73(11):1111–9. 10.1016/j.biopsych.2012.12.015 23332354 PMC3636170

[38] Vollmer LL Strawn JR Sah R: Acid-base dysregulation and chemosensory mechanisms in panic disorder: a translational update. *Transl Psychiatry.* 2015;5:e572. 10.1038/tp.2015.67 26080089 PMC4471296

[39] Riske L Thomas RK Baker GB: Lactate in the brain: an update on its relevance to brain energy, neurons, glia and panic disorder. *Ther Adv Psychopharmacol.* 2017;7(2):85–9. 10.1177/2045125316675579 28255438 PMC5315230

[40] Perna G Dario A Caldirola D: Panic disorder: the role of the balance system. *J Psychiatr Res.* 2001;35(5):279–86. 10.1016/S0022-3956(01)00031-0 11591430

[41] Coelho CM Balaban CD: Visuo-vestibular contributions to anxiety and fear. *Neurosci Biobehav Rev.* 2015;48:148–59. 10.1016/j.neubiorev.2014.10.023 25451199

[42] Teggi R Caldirola D Colombo B: Dizziness, migrainous vertigo and psychiatric disorders. *J Laryngol Otol.* 2010;124(3):285–90. 10.1017/S0022215109991976 19954562

[43] Teggi R Caldirola D Bondi S: Vestibular testing in patients with panic disorder and chronic dizziness. *Acta Otorhinolaryngol Ital.* 2007;27(5):243–7. 18198754 PMC2640032

[44] Perna G Alpini D Caldirola D: Serotonergic modulation of the balance system in panic disorder: an open study. *Depress Anxiety.* 2003;17(2):101–6. 10.1002/da.10092 12621600

[45] Castrogiovanni P Pieraccini F Iapichino S: Electroretinogram B-wave amplitude in panic disorder. *CNS Spectr.* 2001;6(3):210–3. 10.1017/S1092852900008580 16951655

[46] Srinivasan K Ashok MV Vaz M: Decreased chaos of heart rate time series in children of patients with panic disorder. *Depress Anxiety.* 2002;15(4):159–67. 10.1002/da.10046 12112720

[47] Perna G Ieva A Caldirola D: Respiration in children at risk for panic disorder. *Arch Gen Psychiatry.* 2002;59(2):185–6. 10.1001/archpsyc.59.2.185 11825140

[48] Zwanzger P Bradwejn J Diemer J: Differences in saccadic eye movements in subjects at high and low risk for panic disorder. *Curr Pharm Des.* 2012;18(35):5685–90. 10.2174/138161212803530934 22632478

[49] Meuret AE Rosenfield D Wilhelm FH: Do unexpected panic attacks occur spontaneously? *Biol Psychiatry.* 2011;70(10):985–91. 10.1016/j.biopsych.2011.05.027 21783179 PMC3327298

[50] Panksepp J: The basic emotional circuits of mammalian brains: do animals have affective lives? *Neurosci Biobehav Rev.* 2011;35(9):1791–804. 10.1016/j.neubiorev.2011.08.003 21872619

[51] Panksepp J: Affective preclinical modeling of psychiatric disorders: taking imbalanced primal emotional feelings of animals seriously in our search for novel antidepressants. *Dialogues Clin Neurosci.* 2015;17(4):363–79. 26869838 10.31887/DCNS.2015.17.4/jpanksepp PMC4734875

[52] Panksepp J Northoff G: The trans-species core SELF: the emergence of active cultural and neuro-ecological agents through self-related processing within subcortical-cortical midline networks. *Conscious Cogn.* 2009;18(1):193–215. 10.1016/j.concog.2008.03.002 18485741

[53] Parvizi J Damasio A: Consciousness and the brainstem. *Cognition.* 2001;79(1–2):135–60. 10.1016/S0010-0277(00)00127-X 11164026

[54] Perna G Caldirola D Bellodi L: Panic disorder: from respiration to the homeostatic brain. *Acta Neuropsychiatr.* 2004;16(2):57–67. 10.1111/j.0924-2708.2004.0080.x 26983998

[55] Angeli D Ferrell JE Jr Sontag ED: Detection of multistability, bifurcations, and hysteresis in a large class of biological positive-feedback systems. *Proc Natl Acad Sci U S A.* 2004;101(7):1822–7. 10.1073/pnas.0308265100 14766974 PMC357011

[56] Pohl R Yeragani VK Balon R: Isoproterenol-induced panic attacks. *Biol Psychiatry.* 1988;24(8):891–902. 10.1016/0006-3223(88)90224-7 3069135

[57] Levitan MN Nardi AE: Nocturnal panic attacks: clinical features and respiratory connections. *Expert Rev Neurother.* 2009;9(2):245–54. 10.1586/14737175.9.2.245 19210198

[58] Nakamura M Sugiura T Nishida S: Is nocturnal panic a distinct disease category? Comparison of clinical characteristics among patients with primary nocturnal panic, daytime panic, and coexistence of nocturnal and daytime panic. *J Clin Sleep Med.* 2013;9(5):461–7. 10.5664/jcsm.2666 23674937 PMC3629320

[59] Chouchou F Desseilles M: Heart rate variability: a tool to explore the sleeping brain? *Front Neurosci.* 2014;8:402. 10.3389/fnins.2014.00402 25565936 PMC4263095

[60] Malik V Smith D Lee-Chiong T: Respiratory Physiology During Sleep. *Sleep Med Clin.* 2012;7(3):497–505. 10.1016/j.jsmc.2012.06.011

[61] Bouton ME Mineka S Barlow DH: A modern learning theory perspective on the etiology of panic disorder. *Psychol Rev.* 2001;108(1):4–32. 10.1037/0033-295X.108.1.4 11212632

[62] Acheson DT Forsyth JP Moses E: Interoceptive fear conditioning and panic disorder: the role of conditioned stimulus-unconditioned stimulus predictability. *Behav Ther.* 2012;43(1):174–89. 10.1016/j.beth.2011.06.001 22304889

[63] De Cort K Griez E Büchler M: The role of "interoceptive" fear conditioning in the development of panic disorder. *Behav Ther.* 2012;43(1):203–15. 10.1016/j.beth.2011.06.005 22304891

[64] Domschke K Stevens S Pfleiderer B: Interoceptive sensitivity in anxiety and anxiety disorders: an overview and integration of neurobiological findings. *Clin Psychol Rev.* 2010;30(1):1–11. 10.1016/j.cpr.2009.08.008 19751958

[65] Sgoifo A Carnevali L Alfonso Mde L: Autonomic dysfunction and heart rate variability in depression. *Stress.* 2015;18(3):343–52. 10.3109/10253890.2015.1045868 26004818

[66] Bakhshaie J Zvolensky MJ Goodwin RD: Cigarette smoking and the onset and persistence of panic attacks during mid-adulthood in the United States: 1994–2005. *J Clin Psychiatry.* 2016;77(1):e21–4. 10.4088/JCP.14m09290 26845274 PMC11846044

[67] Cosci F Knuts IJ Abrams K: Cigarette smoking and panic: a critical review of the literature. *J Clin Psychiatry.* 2010;71(5):606–15. 10.4088/JCP.08r04523blu 19961810

[68] Isensee B Wittchen HU Stein MB: Smoking increases the risk of panic: findings from a prospective community study. *Arch Gen Psychiatry.* 2003;60(7):692–700. 10.1001/archpsyc.60.7.692 12860773

[69] Perna G Barbini B Cocchi S: 35% CO _2_ challenge in panic and mood disorders. *J Affect Disord.* 1995;33(3):189–94. 10.1016/0165-0327(94)00088-Q 7790671

[70] Cheng YF Leu HB Su CC: Association between panic disorder and risk of atrial fibrillation:a nationwide study. *Psychosom Med.* 2013;75(1):30–5. 10.1097/PSY.0b013e318273393a 23107841

[71] Coryell W: Hypersensitivity to carbon dioxide as a disease-specific trait marker. *Biol Psychiatry.* 1997;41(3):259–63. 10.1016/S0006-3223(97)87457-4 9024948

[72] Coryell W Fyer A Pine D: Aberrant respiratory sensitivity to CO _2_ as a trait of familial panic disorder. *Biol Psychiatry.* 2001;49(7):582–7. 10.1016/S0006-3223(00)01089-1 11297715

[73] Perna G Cocchi S Bertani A: Sensitivity to 35% CO2 in healthy first-degree relatives of patients with panic disorder. *Am J Psychiatry.* 1995;152(4):623–5. 10.1176/ajp.152.4.623 7694916

[74] van Beek N Griez E: Reactivity to a 35% CO _2_ challenge in healthy first-degree relatives of patients with panic disorder. *Biol Psychiatry.* 2000;47(9):830–5. 10.1016/S0006-3223(99)00265-6 10812042

[75] Coryell W Dindo L Fyer A: Onset of spontaneous panic attacks: a prospective study of risk factors. *Psychosom Med.* 2006;68(5):754–7. 10.1097/01.psy.0000232268.00327.b4 17012529

[76] Damasio A: Feelings of emotion and the self. *Ann N Y Acad Sci.* 2003;1001:253–61. 10.1196/annals.1279.014 14625365

[77] Araujo HF Kaplan J Damasio H: Neural correlates of different self domains. *Brain Behav.* 2015;5(12):e00409. 10.1002/brb3.409 26807336 PMC4714646

[78] Damasio A Damasio H Tranel D: Persistence of feelings and sentience after bilateral damage of the insula. *Cereb Cortex.* 2013;23(4):833–46. 10.1093/cercor/bhs077 22473895 PMC3657385

[79] Graeff FG: Translational approach to the pathophysiology of panic disorder: Focus on serotonin and endogenous opioids. *Neurosci Biobehav Rev.* 2017;76(Pt A):48–55. 10.1016/j.neubiorev.2016.10.013 28073587

[80] Graeff FG Sant'Ana AB Vilela-Costa HH: New Findings on the Neurotransmitter Modulation of Defense in the Dorsal Periaqueductal Gray. *CNS Neurol Disord Drug Targets.* 2015;14(8):988–95. 10.2174/1871527314666150909114558 26350338

[81] Schimitel FG Müller CJ Tufik S: Evidence of a suffocation alarm system sensitive to clinically-effective treatments with the panicolytics clonazepam and fluoxetine. *J Psychopharmacol.* 2014;28(12):1184–8. 10.1177/0269881114552714 25277323

[82] Spiacci A Jr Sergio Tde O da Silva GS: Serotonin in the dorsal periaqueductal gray inhibits panic-like defensive behaviors in rats exposed to acute hypoxia. *Neuroscience.* 2015;307:191–8. 10.1016/j.neuroscience.2015.08.045 26319117

[83] Perna G Guerriero G Brambilla P: Panic and the brainstem: clues from neuroimaging studies. *CNS Neurol Disord Drug Targets.* 2014;13(6):1049–56. 10.2174/1871527313666140612112923 24923341

[84] Goossens L Leibold N Peeters R: Brainstem response to hypercapnia: a symptom provocation study into the pathophysiology of panic disorder. *J Psychopharmacol.* 2014;28(5):449–56. 10.1177/0269881114527363 24646808

[85] Šilhán P Jelínková M Walter U: Transcranial sonography of brainstem structures in panic disorder. *Psychiatry Res.* 2015;234(1):137–43. 10.1016/j.pscychresns.2015.09.010 26371456

[86] Sobanski T Wagner G: Functional neuroanatomy in panic disorder: Status quo of the research. *World J Psychiatry.* 2017;7(1):12–33. 10.5498/wjp.v7.i1.12 28401046 PMC5371170

[87] Wiest G Lehner-Baumgartner E Baumgartner C: Panic attacks in an individual with bilateral selective lesions of the amygdala. *Arch Neurol.* 2006;63(12):1798–801. 10.1001/archneur.63.12.1798 17172622

[88] Feinstein JS Buzza C Hurlemann R: Fear and panic in humans with bilateral amygdala damage. *Nat Neurosci.* 2013;16(3):270–2. 10.1038/nn.3323 23377128 PMC3739474

[89] Khalsa SS Feinstein JS Li W: Panic Anxiety in Humans with Bilateral Amygdala Lesions: Pharmacological Induction via Cardiorespiratory Interoceptive Pathways. *J Neurosci.* 2016;36(12):3559–66. 10.1523/JNEUROSCI.4109-15.2016 27013684 PMC4804013

[90] Fukuda K: Novel hypothesis for the cause of panic disorder via the neuroepithelial bodies in the lung. *Med Hypotheses.* 2005;64(6):1192–7. 10.1016/j.mehy.2004.11.037 15823715

[91] Ling L Fuller DD Bach KB: Chronic intermittent hypoxia elicits serotonin-dependent plasticity in the central neural control of breathing. *J Neurosci.* 2001;21(14):5381–8. 11438615 10.1523/JNEUROSCI.21-14-05381.2001 PMC6762841

[92] Ostrowski TD Ostrowski D Hasser EM: Depressed GABA and glutamate synaptic signaling by 5-HT _1A_ receptors in the nucleus tractus solitarii and their role in cardiorespiratory function. *J Neurophysiol.* 2014;111(12):2493–504. 10.1152/jn.00764.2013 24671532 PMC4044435

[93] Halberstadt AL Balaban CD: Selective anterograde tracing of the individual serotonergic and nonserotonergic components of the dorsal raphe nucleus projection to the vestibular nuclei. *Neuroscience.* 2007;147(1):207–23. 10.1016/j.neuroscience.2007.03.049 17507165 PMC2093990

[94] Leibold NK van den Hove DL Esquivel G: The brain acid-base homeostasis and serotonin: A perspective on the use of carbon dioxide as human and rodent experimental model of panic. *Prog Neurobiol.* 2015;129:58–78. 10.1016/j.pneurobio.2015.04.001 25930682

[95] Yeragani VK Rao R Tancer M: Paroxetine decreases respiratory irregularity of linear and nonlinear measures of respiration in patients with panic disorder. A preliminary report. *Neuropsychobiology.* 2004;49(2):53–7. 10.1159/000076410 14981334

[96] Perna G Cogo R Bellodi L: Selective serotonin re-uptake inhibitors beyond psychiatry: are they useful in the treatment of severe, chronic, obstructive pulmonary disease? *Depress Anxiety.* 2004;20(4):203–4. 10.1002/da.20041 15643645

[97] Smoller JW Pollack MH Systrom D: Sertraline effects on dyspnea in patients with obstructive airways disease. *Psychosomatics.* 1998;39(1):24–9. 10.1016/S0033-3182(98)71377-5 9538672

[98] Nascimento I de Melo-Neto VL Valença AM: Medicação antipânico e função pulmonar em pacientes com transtorno de pânico. *Rev psiquiatr clín.* 2009;36(4):123–9. 10.1590/S0101-60832009000400001

[99] Mason M Welsh EJ Smith I: Drug therapy for obstructive sleep apnoea in adults. *Cochrane Database Syst Rev.* 2013; (5): CD003002. 10.1002/14651858.CD003002.pub3 23728641 PMC11623339

[100] Suhara T Sudo Y Yoshida K: Lung as reservoir for antidepressants in pharmacokinetic drug interactions. *Lancet.* 1998;351(9099):332–5. 10.1016/S0140-6736(97)07336-4 9652614

[101] Castro EC Sen P Parks WT: The Role of Serotonin Transporter in Human Lung Development and in Neonatal Lung Disorders. *Can Respir J.* 2017;2017: 9064046. 10.1155/2017/9064046 28316463 PMC5337869

[102] Tucker P Adamson P Miranda R Jr: Paroxetine increases heart rate variability in panic disorder. *J Clin Psychopharmacol.* 1997;17(5):370–6. 10.1097/00004714-199710000-00006 9315988

[103] Yeragani VK Jampala VC Sobelewski E: Effects of paroxetine on heart period variability in patients with panic disorder: a study of holter ECG records. *Neuropsychobiology.* 1999;40(3):124–8. 10.1159/000026608 10494046

[104] Yeragani VK Pohl R Jampala VC: Effects of nortriptyline and paroxetine on QT variability in patients with panic disorder. *Depress Anxiety.* 2000;11(3):126–30. 10.1002/(SICI)1520-6394(2000)11:3<126::AID-DA7>3.0.CO;2-1 10875054

[105] Yeragani VK Rao R: Effect of nortriptyline and paroxetine on measures of chaos of heart rate time series in patients with panic disorder. *J Psychosom Res.* 2003;55(6):507–13. 10.1016/S0022-3999(03)00023-0 14642980

[106] Nezafati MH Eshraghi A Vojdanparast M: Selective serotonin reuptake inhibitors and cardiovascular events: A systematic review. *J Res Med Sci.* 2016;21:66. 10.4103/1735-1995.189647 27904611 PMC5122239

[107] Vaclavik J Krenkova A Kocianova E: 7B.04: Effect of Sertraline in Paroxysmal Hypertension. *J Hypertens.* 2015;33(Suppl 1):e93. 10.1097/01.hjh.0000467601.49032.62 26102972

[108] Pousti A Bakhtiarian A Najafi R: Effect of sertraline on ouabain-induced arrhythmia in isolated guinea-pig atria. *Depress Anxiety.* 2009;26(8):E106–10. 10.1002/da.20407 19242981

[109] Pousti A Malihi G Naghibi B: Effect of citalopram on ouabain-induced arrhythmia in isolated guinea-pig atria. *Hum Psychopharmacol.* 2003;18(2):121–4. 10.1002/hup.446 12590405

[110] Venault P Rudrauf D Lepicard EM: Balance control and posture in anxious mice improved by SSRI treatment. *Neuroreport.* 2001;12(14):3091–4. 10.1097/00001756-200110080-00022 11568643

[111] Meuret AE Wilhelm FH Ritz T: Feedback of end-tidal *p*CO _2_ as a therapeutic approach for panic disorder. *J Psychiatr Res.* 2008;42(1):560–8. 10.1016/j.jpsychires.2007.06.005 17681544 PMC2890048

[112] Tolin DF McGrath PB Hale LR: A Multisite Benchmarking Trial of Capnometry Guided Respiratory Intervention for Panic Disorder in Naturalistic Treatment Settings. *Appl Psychophysiol Biofeedback.* 2017;42(1):51–8. 10.1007/s10484-017-9354-4 28194546 PMC5344940

[113] Broocks A Bandelow B Pekrun G: Comparison of aerobic exercise, clomipramine, and placebo in the treatment of panic disorder. *Am J Psychiatry.* 1998;155(5):603–9. 10.1176/ajp.155.5.603 9585709

[114] Gaudlitz K Plag J Dimeo F: Aerobic exercise training facilitates the effectiveness of cognitive behavioral therapy in panic disorder. *Depress Anxiety.* 2015;32(3):221–8. 10.1002/da.22337 25515221

[115] Bartley CA Hay M Bloch MH: Meta-analysis: aerobic exercise for the treatment of anxiety disorders. *Prog Neuropsychopharmacol Biol Psychiatry.* 2013;45:34–9. 10.1016/j.pnpbp.2013.04.016 23643675

[116] Jacob RG Whitney SL Detweiler-Shostak G: Vestibular rehabilitation for patients with agoraphobia and vestibular dysfunction: a pilot study. *J Anxiety Disord.* 2001;15(1–2):131–46. 10.1016/S0887-6185(00)00047-5 11388356

[117] Sánchez-Meca J Rosa-Alcázar AI Marín-Martínez F: Psychological treatment of panic disorder with or without agoraphobia: a meta-analysis. *Clin Psychol Rev.* 2010;30(1):37–50. 10.1016/j.cpr.2009.08.011 19775792

[118] Perna G Caldirola D: Management of Treatment-Resistant Panic Disorder. *Curr Treat Options Psychiatry.* 2017;4(4):371–86. 10.1007/s40501-017-0128-7 29238651 PMC5717132

